# Functionality and cognition of older adults hospitalized in a university hospital

**DOI:** 10.1590/0034-7167-2025-0054

**Published:** 2026-01-26

**Authors:** Felipe Bueno da Silva, Gustavo Carrijo Barbosa, Luana Aparecida da Rocha, Aline Cristina Martins Gratão

**Affiliations:** I Universidade Federal de São Carlos. São Carlos, São Paulo, Brazil

**Keywords:** Hospitalization, Aged, Health Care Surveys, Health Services for the Aged, Nursing Care., Hospitalización, Anciano, Salud del Anciano, Atención a los Ancianos, Cuidado de Enfermería al Anciano Hospitalizado.

## Abstract

**Objectives:**

to verify the association between functionality, cognitive function, and age group among hospitalized older adults.

**Methods:**

this was a descriptive and cross-sectional study conducted with patients hospitalized in the adult/older adult medical clinic sector between April and September 2023. For the association analysis, Fisher’s exact test and a Poisson regression model with robust variance were used.

**Results:**

high rates of multimorbidity, polypharmacy, probable cognitive deficit, and varying degrees of dependence in performing basic and instrumental activities of daily living were observed in the sample. A higher prevalence of cognitive deficit and dependence in basic activities was found among participants aged 80 years or older.

**Conclusions:**

the relationship between functionality, cognitive function, and age group requires frequent assessments, hospitalization protocols, and discharge planning. Continuous training of the nursing team is also highlighted as essential for identifying complications and promoting the comprehensive health of older patients.

## RESUMEN

**Objetivos:**

verificar la asociación entre funcionalidad, función cognitiva y grupo etario de personas mayores hospitalizadas.

**Métodos:**

estudio descriptivo y transversal realizado con pacientes hospitalizados en el sector de clínica médica adulto/mayores entre abril y septiembre de 2023. Para el análisis de asociación, se emplearon la prueba exacta de Fisher y el modelo de regresión de Poisson con varianza robusta.

**Resultados:**

se observaron altos índices de polipatología, polifarmacia, probable déficit cognitivo y determinado grado de dependencia para la realización de actividades básicas e instrumentales de la vida diaria en la muestra. Se identificó una mayor prevalencia de déficit cognitivo y de dependencia en actividades básicas entre los participantes de 80 años o más.

**Conclusiones:**

la relación entre funcionalidad, función cognitiva y grupo etario requiere evaluaciones frecuentes, protocolos específicos para la hospitalización y una adecuada preparación para el alta hospitalaria. Asimismo, se destaca la importancia de la capacitación continua del equipo de enfermería para identificar complicaciones y promover la salud integral de las personas mayores hospitalizadas.

## INTRODUCTION

Data from the Brazilian Institute of Geography and Statistics (IBGE) indicate that 37.8% of Brazil’s population will be older adults by the year 2070^(1)^. This demographic transformation, however, may pose a significant challenge for health systems that are still unprepared, as there is a growing incidence of non-communicable diseases (NCDs) as life expectancy increases^(2)^. A recent report from the Pan American Health Organization analyzed the burden of morbidity and mortality in the Americas from 2000 to 2019. The data reveal an increase in comorbidities and disabilities resulting from NCDs, driven by the accelerated aging of the population^(2)^.

With the advent of population aging, resulting from demographic transition, it is estimated that healthcare will become more costly due to increased public consumption of hospital care and preventive and curative activities, which are expected to rise both because of the demand for specialized care and the incorporation of new health technologies^(3)^. Older adults represent the age group with the highest demand for services in this area, characterized by higher hospitalization rates and longer hospital stays compared to other age groups. This scenario is largely attributed to the prevalence of NCDs and the complications associated with these conditions^(4,5)^.

Hospitalization constitutes a fundamental resource in the healthcare of older adults, integrating the care network. However, frequent and prolonged hospital stays can generate negative consequences for this population segment. Health demands, especially those related to NCDs, stand out as the main determining factors for hospitalization in this group. Although often indispensable, hospitalizations can be directly associated with risks such as immobility, incontinence, depression, cognitive decline, and reduced functional capacity^(6,7)^. When combined, these conditions are risk factors for premature mortality, prolonged hospitalization, and readmissions^(8)^.

After hospital discharge, it is common to observe worsening functional and cognitive capacity among older adults, which negatively impacts their quality of life and imposes a burden on family members and healthcare professionals responsible for their care^(9)^. This scenario highlights the importance of early identification of these declines to minimize losses and promote the development of strategies that stimulate functional and cognitive rehabilitation, favoring the maintenance of autonomy for these patients even during hospitalization.

## OBJECTIVES

To verify the association between functionality, cognition, and age group among hospitalized older adults.

## METHODS

### Ethical aspects

The present study was submitted to the Research Ethics Committee of the Federal University of São Carlos.

### Study design, period, and setting

This was a descriptive and cross-sectional study, guided by the STROBE tool, and conducted with a non-probabilistic and intentional sample. All stages of the study were carried out in the adult/older adult inpatient unit of a university hospital (UH) located in the interior (Central-East region) of the state of São Paulo. The UH provides services in the areas of emergency care, adult/older adult and pediatric inpatient care, psychosocial care, intensive care unit, surgical center, support services, diagnostics, and therapy. All older adult patients who were hospitalized in the referenced hospital ward between April and September 2023, and whose conditions allowed them to answer the questions, were invited to participate in the study.

### Sample and inclusion and exclusion criteria

Individuals aged 60 years or older, of both sexes, who were hospitalized for clinical or surgical conditions during the data collection period, who were able to answer the questionnaires or had assistance from a family member/caregiver to help them, and who agreed to participate by signing the Informed Consent Form (ICF) or, when necessary, the ICF of the legal guardian, were included. Older adults in isolation or those requiring contact precautions, as well as older adults with clinical conditions that made participation in the study impossible-whether due to the complexity of their clinical condition, communication difficulties, or the need for interventions by the care team-were excluded. To maintain and preserve the overall well-being of older adults, data collection could not be completed for some patients, which also resulted in the exclusion of these participants from the sample.

### Study Protocol

The researcher’s initial contact in the medical clinic unit was established through the presentation of the study to the nurses working in the sector, who indicated the number of hospitalized older adults. Subsequently, contact was made with the participant and their companion through the researcher’s introduction and explanation of the study. The primary approach consisted of the individual application of the assessment protocol, following the participant’s consent, obtained through the signing of the ICF or the legal guardian’s ICF when necessary. At the end of the assessment, the information was supplemented by reviewing the patients’ medical records, made available by the nursing team, in which data regarding diagnosis, comorbidity history, and continuous medication use were confirmed. The assessment protocol consisted of the following items:

- Sociodemographic and health information questionnaire: developed by the researchers, composed of sociodemographic data such as gender, age, marital status, education, date of birth, and skin color, aiming to characterize the participants’ profiles. Additionally, the questionnaire sought to understand each patient’s current and past medical history, including medication use, family history, falls in the past six months, lifestyle habits, presence of polypharmacy (use of ≥5 medications), and multimorbidity (presence of ≥5 morbidities).
- Assessment of dependence level for basic activities of daily living (BADL): conducted using the Katz Index, adapted for Brazil by Lino et al.^(10)^, which addresses issues related to self-care. The result is obtained by summing the number of positive responses, which represent independence. Patients scoring between five and six points are considered independent, those scoring between three and four points are considered partially dependent, and those scoring from zero to two points are considered highly dependent.
- Assessment of dependence level for instrumental activities of daily living (IADL): conducted using the Lawton and Brody scale, adapted for Brazil by Santos and Virtuoso Júnior^(11)^. The score ranges from seven to 21 points, with a score of seven indicating dependence, a score between eight and 20 indicating partial dependence, and 21 points indicating independence. Considering the data collection context, the instrument was adapted to ensure that the results reflected the patients’ reality. The first response option, previously only “Yes”, was modified to “Yes/No due to hospitalization, but capable”, to better address the condition of hospitalized patients.
- Assessment of cognitive performance: conducted using the 10-Point Cognitive Screener (10-CS), developed in Brazil by Apolinario et al.^(12)^. The instrument evaluates cognitive performance through six questions that assess orientation, verbal fluency, and word recall. The test score ranges from zero to ten points. Cognitive function is considered preserved in individuals scoring between eight and ten points. Scores between six and seven points indicate possible cognitive impairment, while scores equal to or less than five suggest probable cognitive impairment.

### Data Analysis and Statistics

The data were entered into a database in Excel^®^ using a double-entry validation process and then imported into SAS 9.4^®^ software. Initially, the data were described using absolute and relative frequencies (qualitative variables) and measures of central tendency and dispersion: mean, standard deviation, minimum, median, and maximum (quantitative variables). To analyze the association between age group and the scales, participants were categorized into two subgroups (60 to 79 years and 80 years or older), and the outcomes were recategorized into binary form (yes or no): dependence according to the Katz scale, dependence according to the Lawton and Brody scale, and probable/possible cognitive deficit according to the 10-CS. To estimate the prevalence ratio, a univariable Poisson regression model with robust variance was used^(13)^. Associations between age group and outcomes were analyzed using Fisher’s exact test. A significance level of 5% (p≤0.05) and a 95% confidence interval were adopted^(14)^.

## RESULTS

During the data collection period, 51 older adults were evaluated in the inpatient unit of the UH and fully completed the assessment protocol, comprising the final sample. As shown in Table 1, most respondents were female (56.9%) and self-identified as White (88.2%). The mean age of the sample was 73.7 years (±9.7), ranging from 60 to 97 years. Regarding marital status, 41.2% of participants were married or had a partner. The mean education level was 7.3 years of schooling (±4.5). Among the patients, 90.2% reported being retired and receiving some type of benefit.

**Table 1:** Sociodemographic distribution of the older adults evaluated, São Carlos, São Paulo, Brazil, 2025

Sociodemographic Characteristics	n (%)
**Gender**	
Female	29 (56.9)
Male	22 (43.1)
**Marital Status**	
Married/With partner	21 (41.2)
Widowed	14 (27.4)
Divorced/Separated	08 (15.7)
Single	08 (15.7)
**Skin Color**	
White	45 (88.2)
Brown	03 (5.9)
Black	03 (5.9)
**Age Group**	
60 to 69 years	21 (41.2)
70 to 79 years	16 (31.4)
80 to 89 years	10 (19.6)
90 years or older	04 (7.8)
**Type of Benefit**	
Retirement	46 (90.2)
Does not receive	04 (7.8)
Pensioner	01 (2)

### Health characteristics and hospitalization information

In Chart 1, it is observed that 60.8% of the patients were in a condition of multimorbidity, with a mean of 11.09 comorbidities (±21.7). Among the main reported health conditions, hypertension (66.7%), diabetes mellitus (54.9%), and cardiovascular diseases (50.9%) were the most prevalent. Daily medication use was reported by 98% of the sample, with 62.7% classified as experiencing polypharmacy, with a mean of 5.53 medications (±2.68). Regarding sleep quality, 35.3% of the patients reported suffering from insomnia. In the past six months, 58.8% of the patients reported having experienced falls. Skin abrasions were the most frequently mentioned consequence among those who fell (50%).

**Table 2:** Information regarding cognitive function and level of dependence in basic and instrumental activities of daily living among the older adults evaluated, São Carlos, São Paulo, Brazil, 2025

Variables	n (%) / Mean (SD)
**10-Point Cognitive Screener**	4.2 (2.76)
Normal cognitive function	07 (13.7)
Possible cognitive deficit	12 (23.5)
Probable cognitive deficit	32 (62.7)
**Katz Index**	3.65 (2.53)
Independent	22 (43.1)
Partially dependente	09 (17.7)
Dependent	20 (39.2)
**Lawton and Brody Scale**	13.02 (5.26)
Independent	04 (7.9)
Partially dependent	38 (74.5)
Dependent	09 (17.6)

**Chart 1:** Health characteristics and hospitalization information of the older adults evaluated, São Carlos, São Paulo, Brazil, 2025

	n (%)
**Health Characteristics**	
**Number of Morbidities**	
5 or more morbidities	31 (60.8)
3 to 4 morbidities	18 (35.3)
Up to 2 morbidities	02 (3.9)
**Polypharmacy**	
Yes (5 or more medications)	32 (62.7)
No (less than 5 medications)	19 (37.2)
**Sleep Quality**	
No alterations	27 (52.9)
Insomnia	18 (35.2)
Hypersomnia	0 (0)
Other	6 (11.8)
**Falls in the last 6 months**	
Yes	30 (58.8)
No	21 (41.2)
**Number of falls**	
1 fall	23 (76.7)
2 or more	07 (23.3)
**Impact of the fall**	
Abrasions	15 (50)
Hospitalizations	06 (20)
Fear	05 (16.7)
Fracture	04 (13.3)
**Circumstances of the fall**	
Muscle weakness	20 (66.7)
Trip/slip	06 (20)
Acute illness	03 (10)
Unconsciousness	01 (3.3)
**Hospitalization Characteristics**	
**Main Complaint**	
Dyspnea	11 (21.6)
Stroke	05 (9.8)
Congestive Heart Failure	05 (9.8)
Chronic Kidney Disease	05 (9.8)
Pneumonia	04 (7.8)
Hepatic Cirrhosis	03 (5.9)
Other complaints	18 (35.3)
**Reason for Hospitalization**	
Respiratory system diseases	17 (33.3)
Cardiovascular diseases	09 (17.6)
Digestive system diseases	08 (15.7)
Genitourinary system diseases	07 (13.7)
Neurodegenerative diseases	06 (11.8)
Infectious and parasitic diseases	01 (2)
Other	03 (5.9)
**Hospitalization ICD**	
J18 (Pneumonia)	11 (21.57)
K74 (Hepatic fibrosis and cirrhosis)	07 (13.73)
I50 (Heart failure)	06 (11.76)
I64 (Stroke)	05 (9.8)
N17 (Acute renal failure)	05 (9.8)
**Type of hospitalization**	
Clinical	50 (98)
Surgical	01 (2)

Muscle weakness was identified as one of the main factors associated with falls, present in 66.7% of cases. The primary complaint among hospitalized participants was dyspnea (21.6%), followed by complications from stroke (cerebrovascular accident - CVA), congestive heart failure (CHF), and chronic kidney disease (CKD), each accounting for 9.8%. Regarding the reason for hospitalization, categorized by physiological systems, respiratory diseases were the most prevalent in the sample (33.3%), followed by cardiovascular diseases (17.6%). Clinical hospitalization was predominant, covering 98% of cases. The main hospitalization diagnosis, according to ICD-10, was identified in the patients’ medical records, with the most common being pneumonia of unspecified etiology (J18) (21.6%) and hepatic fibrosis and cirrhosis (K74) (13.73%).

### Assessment Scales

When evaluating the patients’ cognitive function, a mean score of 4.2 points (±2.7) was observed, with a predominance of probable cognitive deficit (62.7%). For the level of dependence in BADL, the mean score was 3.6 points (±2.5), with independence being the most prevalent condition (43.1%). For the level of dependence in IADL, the mean score was 13.2 points (±5.2), with partial dependence predominating (74.5%) for this type of activity. Detailed information is presented in Table 2.

### Association between age group, cognitive decline, and functional dependence

After applying Fisher’s exact test, it was possible to identify the presence or absence of an association between age group and the outcomes, considering two groups: older adults aged 60 to 79 years (n=37) and those aged 80 years or older (n=14). The analysis showed a statistically significant association, of moderate magnitude, between age group and the variables cognitive function (p<0.02), BADL (p<0.01), and IADL (p<0.03). Greater dependence in BADL and poorer cognitive performance were observed with advancing age. For IADL, a more pronounced partial dependence was identified among individuals under 80 years of age, along with a gradual increase in dependence as age advanced. Detailed information is presented in Table 3.

**Table 3:** Association between age group, cognitive function, and level of dependence for performing activities of daily living among the older adults evaluated, São Carlos, São Paulo, Brazil, 2025

Variables	Age group		*p* value^*^
	60 to 79 years (n=37)	≥80 years (n=14)	
Cognitive performance			<0.02
Normal cognitive function	7 (18.9%)	0 (0%)
Possible cognitive deficit	11 (29.7%)	01 (7.14%)
Probable cognitive deficit	19 (51.3%)	13 (92.86%)
**Dependence in BADL**			<0.01
Independent	21 (56.7%)	01 (7.1%)	
Partially dependente	6 (16.2%)	03 (21.4%)	
Dependent	10 (27%)	10 (71.4%)	
**Dependence in IADL**			
Independent	4 (10.8%)	05 (35.7%)	<0.03
Partially dependente	31 (83.8%)	07 (50%)	
Dependent	2 (5.4%)	02 (14.3%)	

BADL - Basic Activities of Daily Living; IADL - Instrumental Activities of Daily Living;

* Fisher’s exact test.

The estimation of the prevalence ratio between the age group variable and the outcomes, performed using the Poisson regression model, is presented in Table 4. The association between age group and BADL shows that, among participants aged 80 years or older, the prevalence of some degree of dependence is approximately twice as high compared to older adults under 80 years of age. Regarding cognitive assessment, it is estimated that, on average, the prevalence of possible/probable cognitive deficit is 23% higher among the age group of 80 years or older. No associations were observed between age group and IADL.

**Table 4:** Associations between age group and the outcomes recategorized in a binary model, São Carlos, São Paulo, Brazil, 2025

Variable	Age group		Prevalence Ratio^*^	95% Confidence Interval		*p* value
	<80 years (n=37)	≥80 years (n=14)				
Some dependence in BADL						
No	21 (56.76%)	01 (7.14%)	2.15	1.44	3.19	<0.01
Yes	16 (43.24%)	13 (92.86%)				
Some dependence in IADL						
No	2 (5.41%)	02 (14.29%)	0.91	0.72	1.14	0.40
Yes	35 (94.59%)	12 (85.71%)				
Probable or possible cognitive deficit						
No	7 (18.92%)	0 (0%)	1.23	1.06	1.44	0.01
Yes	30 (81.08%)	14 (100%)				

BADL - Basic Activities of Daily Living; IADL - Instrumental Activities of Daily Living;

* Estimated using the Poisson regression model with robust variance.

## DISCUSSION

The findings of this study show a predominance of female patients in the inpatient unit, corroborating the results of other studies conducted in the context of clinical hospitalization^(15)^. This phenomenon may be attributed to the greater participation of women in health-related research, due to their more frequent exposure to treatments throughout life and the feminization process of aging^(16)^. In other studies conducted in hospital settings, a predominance of male patients has been observed. This trend may be related to men’s tendency to seek medical care and services at later stages, making them more vulnerable to hospitalization due to the progression and worsening of their health conditions^(17)^.

The mean age of the participants was 73.6 years, with 41% of the sample belonging to the 60-69 age group. Studies investigating the health behaviors of older adults in hospital environments report mean ages ranging between 70 and 80 years^(5,18)^. It is already well established in the national literature that both the number of hospitalizations and the mortality rate increase concomitantly with the analyzed age group^(19)^.

In the present study, the average years of schooling were 7.35, indicating a predominance of low education levels among participants, which aligns with other studies that have identified low education levels among older adults in the context of hospitalization^(20,21)^. Low levels of health literacy are associated with higher rates of hospitalization and mortality, as well as poor adherence to treatment regimens and reduced effectiveness of preventive measures, significantly impacting the quality of life of this population^(22)^. These factors are also related to social exclusion, limited access to information, and unfavorable socioeconomic conditions^(23)^. This situation may be attributed to the absence of protective factors, such as the ability to read and write, and the loss of important information about health and self-care, which favors exposure to misinformation^(22)^.

Regarding current occupation and income source, most participants were retired (90.2%). In a systematic literature review, Melo, Falsarella, and Neri^(24)^ highlighted income and education as key factors associated with frailty in older adults. Given the impact of these factors on the quality of life of these individuals, public investment in effective strategies is necessary, such as expanding access to formal education adapted to the needs of the older population, aiming to promote healthy aging, autonomy, and social integration, thereby contributing to physical and mental health, reducing social isolation, and increasing the sense of purpose^(25)^.

Regarding health-related issues, the presence of multimorbidity was observed in 60.8% of the sample. A study conducted by Tavares and Dias^(26)^, with a sample of 2,142 older adults, revealed that 98.3% of the participants had between four and seven distinct health conditions. The presence of these conditions can complicate treatment and significantly increase the risk of complications during hospitalization, prolong the hospital stay, and raise the risk of post-hospitalization complications, highlighting the need to integrate different levels of healthcare to ensure follow-up and early identification of adverse outcomes in these individuals^(27)^. The literature shows an association between chronic diseases such as cancer, diabetes mellitus, cardiovascular disorders, and pulmonary diseases, which often lead to excessive medication use and the need for comprehensive strategies for medication management^(28,29)^.

The prevalence of polypharmacy was observed in 62.7% of the sample, reflecting the national trend of using five or more daily medications^(30)^. Chang et al.^(31)^ conducted a comprehensive study with more than 3 million individuals over the age of 65, investigating the correlation between polypharmacy and the risks of hospitalization and mortality. The results demonstrated a growing positive association between the number of prescribed medications and the likelihood of adverse events. The negative effects of complex medication regimens on clinical outcomes were evidenced in the investigation of the relationship between polypharmacy, dementia, and hospital mortality in patients^(32)^. Therefore, polypharmacy can trigger severe consequences such as falls and cognitive deterioration, often leading to hospitalization^(33)^.

The occurrence of falls in the past six months was reported by 58.8% of the sample, with muscle weakness identified as the main cause (66.7%). The reduction in functional capacity, combined with physiological changes associated with aging, increases the prevalence of falls in this population^(34)^. Additionally, medication use is considered a risk factor for falls, especially due to polypharmacy and resulting drug interactions^(35)^. According to Sugitani and Ito^(36)^, medications such as diuretics and antihypertensives, frequently prescribed for the treatment of systemic arterial hypertension, are associated with an increased risk of falls due to postural hypotension and dizziness. Furthermore, psychotropics, including benzodiazepines, antidepressants, and antipsychotics, contribute to the risk of falls due to their sedative effects and balance impairment^(37)^. Therefore, it is essential to promote health education for patients and their caregivers to ensure the safe use of medications.

Regarding cognitive assessment, a predominance of patients with probable cognitive deficit (62.7%) was found. Cognitive decline is a process that involves changes in intellectual abilities and, as it progresses, older adults face difficulties in performing BADL and IADL, which negatively affects their autonomy, contributes to increased institutionalization rates, and raises the risk of frailty^(38)^. According to Cunha et al.^(39),^ 35% of older adults experience a decline in cognitive capacity when hospitalized. The hospital environment can trigger or intensify cognitive decline in this population due to factors such as polypharmacy, infections, multimorbidities, sensory deprivation, and changes in daily routines^(7)^.

Older adults with low education levels are 4.5 to 5 times more likely to develop some form of cognitive impairment compared to those with higher education levels^(7)^. Additionally, illiterate older adults have a fivefold greater susceptibility to functional dependence. This high rate reflects the lack of access to the educational system in the past, which currently has a significant impact on the cognitive profile of this population^(40)^.

In the present study, a significant correlation between cognitive function and age was observed. It is estimated that, on average, among older adults aged 80 years or older, the prevalence of probable or possible cognitive deficit is 23% higher compared to those under 80 years of age^(41)^. When associated with advanced age and the hospitalization period, Covinsky et al.^(42)^ demonstrated that more than 35% of participants (2,761 individuals aged 70 years or older) experienced a decline in functional capacity. Cunha et al.^(39)^ argue that age is not as relevant as clinical deterioration, which is the main factor of interest, as it contributes to the progression of cognitive decline and impacts the autonomy of this population.

It is crucial that the multidisciplinary team in healthcare services, especially in inpatient settings, implement strategies for cognitive assessment, using specific instruments to identify early signs of cognitive decline and prevent the loss of autonomy, given that the quality of life of these individuals is closely linked to their level of independence in performing daily activities^(43)^. Regarding autonomy for performing BADL, 39.22% of the sample showed dependence, corroborating the findings of other studies^(44)^.

Participants’ age and the degree of dependence in BADL were statistically related. Various factors may contribute to the loss of capacity in hospitalized older adults, such as the development of infections, prolonged hospital stays, and cognitive decline. Additionally, bed rest causes changes in muscle and bone mass, resulting in limitations in performing daily activities^(45)^. A study conducted with older adults hospitalized at the *Hospital das Clínicas* of the Botucatu Medical School identified that the sample’s functional capacity at hospital discharge was lower than that observed before hospitalization^(23)^. Functional losses in BADL are nearly twice as frequent compared to IADL, suggesting that BADL functions as sensitive markers for assessing socialization in this population. Thus, if an individual faces difficulties in any daily living activity, it is likely that they also experience difficulties in two or more IADLs^(46)^.

For IADL, 74.5% of the sample showed partial dependence on this type of activity. In a study with 94 older adults hospitalized in a medical clinic, it was found that, despite preserved autonomy, participants exhibited some degree of dependence in both BADL and IADL, indicating some compromise of independence. Most of the sample was assessed during the first week of hospitalization, suggesting that the patients’ functional status was already compromised before admission, possibly due to exposure to risk factors such as advanced age^(44)^.

For successful aging, it is crucial that older adults maintain not only an adequate multidimensional interaction between physical health, mental health, and social integration but also independence in daily activities. The healthcare team plays a vital role in maintaining the overall functionality of this population, both in the early identification of functional decline and in the implementation of actions to preserve autonomy during the post-hospitalization period^(47)^. This includes, for example, a responsible hospital discharge plan and continuous follow-up at other levels of healthcare.

The implementation of actions aimed at preserving the functionality and cognition of the older population is fundamental in the context of hospitalization. For this purpose, it is essential that nursing professionals possess both theoretical and practical knowledge of the physiological changes associated with aging, allowing not only the early identification of deviations but also the execution of assertive interventions^(48)^. The need for nurses to promote the continuous training of the healthcare team is emphasized, ensuring care that is based on sensitivity, safety, and technical competence. This includes the development of standardized protocols for the assessment of functional decline, combined with targeted actions that encompass both early detection and specialized management of this population.

Among the most effective strategies, continuous assessment of patients’ functional status stands out, as this preservation is closely linked to a reduced risk of mortality in hospitalized older adults^(48)^. Interventions such as the promotion of adapted physical exercises, encouragement of mobility, cognitive stimulation, appropriate medication management, and the provision of individualized care are examples of approaches carried out by multidisciplinary teams in the hospital setting, which have shown positive results and a significant impact on maintaining these factors^(49)^.

### Study limitations

The results of this study should be interpreted in light of certain limitations. Bedside data collection presented significant challenges. Although this methodology offers the advantage of direct and immediate contact with the patient and their clinical reality, difficulties arose related to low education levels, severe cognitive deficits, and debilitating clinical conditions. Additionally, the inability of some participants to communicate, combined with the absence of a caregiver or family member to assist them, also made it impossible to continue data collection at certain times. Moreover, the hospital environment, by its dynamic nature, is subject to emergencies, interruptions, and variations in patients’ health status.

### Contributions to Nursing, Health, or Public Policy

Given the demographic and epidemiological changes characterized by the increase in NCDs and multimorbidities, nursing professionals play a central role not only in direct care but also in identifying hospitalization patterns and articulating strategies to address the complexities of this scenario. The high prevalence of multimorbidity and polypharmacy observed highlights the need for more complex clinical management strategies, in which nursing acts as a mediator between biological, social, and educational demands. The safe management of multiple medications, the prevention of adverse interactions, and the education of patients and families on therapeutic adherence are competencies that require specialized clinical protocols and an interdisciplinary approach. Furthermore, socioeconomic factors, such as limited access to healthcare services and low education levels, demand that nursing incorporate culturally sensitive practices, ensuring that interventions go beyond the hospital environment and align with the realities experienced by patients.

The observed cognitive decline and functional dependence underscore the importance of rehabilitation strategies to maintain the autonomy and quality of life of the older population during hospitalization, reinforcing the urgency of early rehabilitation strategies during this period. The promotion of early mobilization, encouragement of participation in daily life activities, and adaptation of the hospital environment to the functional needs of individuals are essential measures to preserve their quality of life and reduce complications associated with prolonged immobilization. These should be implemented daily by the nursing team.

The older population represents a significant portion of total hospital admissions; therefore, identifying the hospitalization profile and the types of relationships evidenced here allows for the development of new care strategies and perspectives, contributing to the formulation of new health policies and the development of evidence-based health protocols, considering the demands imposed by changes in the Brazilian demographic and epidemiological scenario.

## CONCLUSIONS

The findings reveal a predominance of participants with probable cognitive deficit, independence in BADL, and partial dependence in IADL. The relationship between cognitive decline and functional dependence in daily living activities with advancing age highlights the importance of early and recurrent interventions and assessments. It is essential to implement strategies aimed at maintaining functionality, preventing cognitive decline, and providing psychosocial support, both during hospitalization and in discharge planning, through a structured network that enables follow-up for this population across different levels of healthcare. In addition, continuous training of the multidisciplinary team and caregivers is necessary, contributing to the reduction of readmission rates.

## Data Availability

The research data are available within the article.
