# Supplementary material for: Functionality and cognition of older adults hospitalized in a university hospital
Source: Rev Bras Enferm. 2026 Jan 26;78(6):e20250054. doi: 10.1590/0034-7167-2025-0054 (PMC12854262; doi:10.1590/0034-7167-2025-0054)
Supplement: Translation

# Translation: Funcionalidade e cognição de pessoas idosas internadas em um hospital universitário

**DOI:** 10.1590/0034-7167-2025-0054pt

**Authors:** Felipe Bueno da Silva, Gustavo Carrijo Barbosa, Luana Aparecida da Rocha, Aline Cristina Martins Gratão

**Affiliations:** I Universidade Federal de São Carlos. São Carlos, São Paulo, Brasil

**Keywords:** Hospitalização, Idoso, Saúde do Idoso, Assistência a Idosos, Cuidado de Enfermagem ao Idoso Hospitalizado.

## Abstract

**Objetivos:**

verificar a associação entre funcionalidade, função cognitiva e faixa etária de pessoas idosas hospitalizadas.

**Métodos:**

estudo descritivo e transversal conduzido junto a pacientes hospitalizados no setor de clínica médica adulto/idoso entre abril e setembro de 2023. Para a análise de associação, foram utilizados o teste exato de Fisher e o modelo de regressão de Poisson, com variância robusta.

**Resultados:**

foram observados elevados índices de polipatologia, polifarmácia, provável déficit cognitivo e determinado grau de dependência para a realização de atividades básicas e instrumentais de vida diária entre a amostra. Observou-se maior prevalência de déficit cognitivo e dependência em atividades básicas entre participantes com 80 anos ou mais.

**Conclusões:**

a relação entre funcionalidade, função cognitiva e faixa etária exige avaliações frequentes, protocolos de internação e preparação para alta hospitalar. Destaca-se, também, a importância da capacitação contínua da equipe de enfermagem para identificar complicações e promover a saúde integral do paciente idoso.

## INTRODUÇÃO

Dados do Instituto Brasileiro de Geografia e Estatística (IBGE) apontam que 37,8% dos habitantes do Brasil serão pessoas idosas até o ano de 2070^(1)^. Essa transformação demográfica, no entanto, pode representar um desafio significativo para sistemas de saúde ainda despreparados, pois observa-se a incidência crescente de Doenças Crônicas Não Transmissíveis (DCNTs) à medida que a expectativa de vida aumenta^(2)^. Um relatório recente da Organização Pan-Americana da Saúde analisou a carga de morbidade e mortalidade nas Américas no período de 2000 a 2019. Os dados revelam um aumento nas comorbidades e deficiências decorrentes de DCNTs, impulsionado pelo envelhecimento acelerado da população^(2)^.

Com o advento do envelhecimento populacional, resultante da transição demográfica, estima-se que a assistência à saúde se tornará mais onerosa pelo maior consumo público de cuidados hospitalares e atividades preventivas e curativas, que devem se elevar tanto pela demanda por assistência especializada quanto pela incorporação de novas tecnologias em saúde^(3)^. A população idosa representa o grupo etário com a maior demanda por serviços nessa área, caracterizando-se por taxas de internação elevadas e períodos de permanência hospitalar mais prolongados em comparação às demais faixas etárias. Esse cenário é, em grande parte, atribuído à prevalência das DCNTs e às complicações associadas a essas condições^(4,5)^.

A internação hospitalar constitui um recurso fundamental na assistência à saúde de pessoas idosas, integrando a rede de cuidados. No entanto, internações frequentes e prolongadas podem gerar consequências negativas para essa parcela da população. As demandas de saúde, especialmente relacionadas às DCNTs, destacam-se como os principais fatores determinantes para a hospitalização nesse grupo. Embora muitas vezes indispensáveis, as hospitalizações podem estar diretamente associadas a riscos como imobilidade, incontinência, depressão, declínio cognitivo e redução da capacidade funcional^(6,7)^. Quando associadas, essas condições são fatores de risco para a mortalidade precoce, prolongamento da hospitalização e readmissões^(8)^.

Após a alta hospitalar, é comum observar um agravamento da capacidade funcional e cognitiva entre pessoas idosas, o que impacta negativamente sua qualidade de vida e impõe uma sobrecarga aos familiares e profissionais de saúde responsáveis pelo cuidado^(9)^. Esse cenário destaca a importância da identificação precoce desses declínios, a fim de minimizar as perdas e promover o desenvolvimento de estratégias que estimulem a reabilitação funcional e cognitiva, favorecendo a manutenção da autonomia desses pacientes, mesmo durante o período de internação.

## OBJETIVOS

Verificar a associação entre funcionalidade, cognição e faixa etária de pessoas idosas hospitalizadas.

## MÉTODOS

### Aspectos éticos

O projeto do presente estudo foi submetido ao Comitê de Ética em Pesquisa da Universidade Federal de São Carlos.

### Desenho, período e local do estudo

Trata-se de uma pesquisa descritiva e transversal, norteada pela ferramenta STROBE e conduzida junto a uma amostra não probabilística e intencional. Todas as etapas do estudo foram desenvolvidas na ala de internação do adulto/idoso de um Hospital Universitário (HU) localizado no interior (região Centro-Leste) do estado de São Paulo. O HU atua nas áreas de pronto atendimento, internações adulto/idoso e pediátrica, atenção psicossocial, unidade de terapia intensiva, centro cirúrgico, serviço de apoio, diagnóstico e terapia. Todos os pacientes idosos que estiveram internados na enfermaria hospitalar em questão no período entre abril e setembro de 2023, em condições que permitissem que respondessem às perguntas, foram convidados a participar da pesquisa.

### Amostra e critérios de inclusão e exclusão

Foram incluídos indivíduos com 60 anos ou mais; de ambos os sexos; que estiveram internados por condições clínicas ou cirúrgicas durante o período da coleta de dados; que conseguiram responder aos questionários ou contaram com a assistência de um familiar/cuidador para auxiliá-los, concordando com o Termo de Consentimento Livre e Esclarecido (TCLE) ou, quando necessário, com o TCLE do responsável legal. Foram excluídas as pessoas idosas em isolamento ou que necessitassem de precaução de contato; e pessoas idosas que apresentavam condições clínicas que impossibilitassem sua participação na pesquisa, seja em razão da complexidade do quadro clínico, das dificuldades de comunicação ou da necessidade de intervenções pela equipe assistencial. Visando à manutenção e preservação do bem-estar geral das pessoas idosas, a coleta de dados não pôde ser concluída para alguns pacientes, o que também resultou na exclusão desses participantes da amostra.

### Protocolo do estudo

O contato inicial do pesquisador no setor de clínica médica foi estabelecido por meio da apresentação da pesquisa aos enfermeiros atuantes, os quais sinalizavam a quantidade de pessoas idosas internadas. Em seguida, foi estabelecido contato com o participante e seu acompanhante por meio da apresentação do pesquisador e de seu estudo. A principal abordagem consistiu na aplicação individual do protocolo de avaliação, após a obtenção do consentimento do participante, mediante assinatura do TCLE ou do TCLE para o responsável legal, quando necessário. Ao término da avaliação, as informações foram complementadas por meio da consulta aos prontuários dos pacientes, disponibilizados pela equipe de enfermagem do setor, nos quais se confirmavam dados referentes ao diagnóstico, histórico de comorbidades e uso de medicações contínuas. O protocolo de avaliação foi composto pelos seguintes itens:

- Questionário de caracterização sociodemográfica e informações de saúde: desenvolvido pelos pesquisadores, composto por dados sociodemográficos como gênero, idade, estado civil, escolaridade, data de nascimento, cor da pele e escolaridade, a fim de verificar as características do perfil. Além disso, o questionário buscou compreender a história pessoal atual e pregressa de cada paciente, incluindo uso de medicamentos, histórico familiar, quedas nos últimos seis meses, hábitos de vida, presença de polifarmácia (uso de ≥5 medicamentos) e de polipatologia (presença de ≥5 morbidades).
- Avaliação do grau de dependência para atividades básicas de vida diária (ABVD): realizada por meio da Escala de Katz, adaptada para o Brasil por Lino et al.^(10)^, que aborda questões relacionadas ao autocuidado. O resultado é obtido pelo somatório da quantidade de respostas positivas, que representam independência. É considerado independente o paciente que obtiver entre cinco e seis pontos, parcialmente dependente entre três e quatro pontos e altamente dependente caso pontue de zero a dois.
- Avaliação do grau de dependência para atividades instrumentais de vida diária (AIVD): utilizada a escala de Lawton e Brody, adaptada para o Brasil por Santos e Virtuoso Júnior^(11)^. O escore varia entre sete e 21 pontos, sendo considerado dependente o indivíduo que pontuar sete, parcialmente dependente entre oito e 20 e independente caso some 21 pontos. Tendo em vista o contexto da coleta de dados, o instrumento foi adaptado para garantir que os resultados refletissem a realidade dos pacientes. A primeira resposta do instrumento, anteriormente apenas “Sim”, foi modificada para “Sim/Não devido à internação, mas capaz”, a fim de contemplar melhor as condições dos pacientes internados.
- Avaliação do desempenho cognitivo: realizada por meio do 10-*Point Cognitive Screener* (10-CS), desenvolvido no Brasil por Apolinario et al.^(12)^. O instrumento determina o desempenho cognitivo por meio de seis questões que avaliam a orientação, fluência verbal e recordação de palavras. A pontuação no teste varia entre zero e dez pontos. Considera-se preservada a função cognitiva de indivíduos que obtêm escores entre oito e dez pontos. Por outro lado, escores situados entre seis e sete pontos indicam possível déficit cognitivo, ao passo que valores iguais ou inferiores a cinco sugerem provável déficit cognitivo.

### Análise dos resultados e estatística

Os dados foram tabulados em um banco no aplicativo *Excel* ^®^ pelo processo de validação por dupla entrada e, em seguida, importados para o Software SAS 9.4^®^. Inicialmente, os dados foram descritos por meio de frequências absolutas e frequências relativas (variáveis qualitativas) e por medidas de centralidade e dispersão: média, desvio padrão, mínimo, mediana e máximo (variáveis quantitativas). Para analisar a associação entre a faixa etária e as escalas, os participantes foram categorizados em dois subgrupos (grupo de 60 a 79 anos e grupo com 80 anos ou mais), e os desfechos foram recategorizados de forma binária (sim e não): dependência pela escala Katz, dependência pela escala de Lawton e Brody e provável/possível déficit cognitivo pelo 10-CS. Para estimar a razão de prevalência, foi utilizado o modelo de regressão de Poisson com variância robusta univariável^(13)^. As associações entre faixa etária e os desfechos foram analisadas utilizando o teste exato de Fisher. Adotou-se um nível de significância de 5% (p≤0,05) e intervalo de confiança de 95%^(14)^.

## RESULTADOS

No período da coleta de dados, 51 pessoas idosas foram avaliadas no setor de internação do HU e responderam completamente ao protocolo de avaliação, compreendendo a amostra final. Conforme apresentado na Tabela 1, a maior parte dos entrevistados era do sexo feminino (56,9%) e se autodeclarou como branca (88,2%). A média de idade da amostra foi de 73,7 anos (±9,7), variando entre 60 e 97 anos. Quanto ao estado civil, 41,2% dos participantes eram casados ou possuíam companheiro(a). A média de escolaridade foi de 7,3 anos de estudo (±4,5). Entre os pacientes, 90,2% declararam ser aposentados, recebendo algum tipo de benefício.

**Tabela 1:** Distribuição sociodemográfica das pessoas idosas avaliadas, São Carlos, São Paulo, Brasil, 2025

Características sociodemográficas	n (%)
**Gênero**	
Feminino	29 (56,9)
Masculino	22 (43,1)
**Estado Civil**	
Casado/Com companheiro	21 (41,2)
Viúvo	14 (27,4)
Divorciado/Separado	08 (15,7)
Solteiro	08 (15,7)
**Cor da pele**	
Branca	45 (88,2)
Parda	03 (5,9)
Preta	03 (5,9)
**Faixa etária**	
60 a 69 anos	21 (41,2)
70 a 79 anos	16 (31,4)
80 a 89 anos	10 (19,6)
90 anos ou mais	04 (7,8)
**Tipo de benefício**	
Aposentadoria	46 (90,2)
Não recebe	04 (7,8)
Pensionista	01 (2)

### Características de saúde e informações sobre a internação

No Quadro 1, observa-se que 60,8% dos pacientes estavam em situação de polipatologia, com média de 11,09 comorbidades (±21,7). Entre as principais condições de saúde relatadas, destacam-se a hipertensão arterial (66,7%), diabetes mellitus (54,9%) e doenças cardiovasculares (50,9%). O uso de medicações diárias foi relatado por 98% da amostra, sendo que 62,7% estavam em polifarmácia, com média de 5,53 medicamentos (±2,68). Em relação à qualidade do sono, 35,3% dos pacientes relataram sofrer de insônia. No último semestre, 58,8% dos pacientes relataram ter sofrido quedas. As escoriações foram a consequência mais mencionada entre aqueles que caíram (50%).

**Tabela 2:** Informações referentes à função cognitiva e grau de dependência em atividades básicas e instrumentais de vida diária entre as pessoas idosas avaliadas, São Carlos, São Paulo, Brasil, 2025

Variáveis	n (%) / Média (DP)
**10-*Point Cognitive Screener***	4,2 (2,76)
Função cognitiva normal	07 (13,7)
Possível déficit cognitivo	12 (23,5)
Provável déficit cognitivo	32 (62,7)
**Escala de Katz**	3,65 (2,53)
Independente	22 (43,1)
Parcialmente dependente	09 (17,7)
Dependente	20 (39,2)
**Escala de Lawton e Brody**	13,02 (5,26)
Independente	04 (7,9)
Parcialmente dependente	38 (74,5)
Dependente	09 (17,6)

**Quadro 1:** Características de saúde e informações referentes à internação das pessoas idosas avaliadas, São Carlos, São Paulo, Brasil, 2025

	n (%)
**Características de Saúde**	
**Número de morbidades**	
5 ou mais morbidades	31 (60,8)
3 a 4 morbidades	18 (35,3)
Até 2 morbidades	02 (3,9)
**Polifarmácia**	
Sim (5 ou mais medicamentos)	32 (62,7)
Não (menos que 5 medicamentos)	19 (37,2)
**Qualidade do sono**	
Sem alterações	27 (52,9)
Insônia	18 (35,2)
Hipersonia	0 (0)
Outro	6 (11,8)
**Queda nos últimos 6 meses**	
Sim	30 (58,8)
Não	21 (41,2)
**Número de quedas**	
1 queda	23 (76,7)
2 ou mais	07 (23,3)
**Repercussão da queda**	
Escoriações	15 (50)
Internações	06 (20)
Medo	05 (16,7)
Fratura	04 (13,3)
**Circunstâncias da queda**	
Fraqueza muscular	20 (66,7)
Tropeço/escorregão	06 (20)
Doença aguda	03 (10)
Inconsciência	01 (3,3)
**Características da internação**	
**Queixa principal**	
Dispneia	11 (21,6)
Acidente Vascular Cerebral	05 (9,8)
Insuficiência Cardíaca Congestiva	05 (9,8)
Doença Renal Crônica	05 (9,8)
Pneumonia	04 (7,8)
Cirrose hepática	03 (5,9)
Outras queixas	18 (35,3)
**Motivo da internação**	
Doenças do aparelho respiratório	17 (33,3)
Doenças cardiovasculares	09 (17,6)
Doenças do aparelho digestivo	08 (15,7)
Doenças do aparelho geniturinário	07 (13,7)
Doenças neurodegenerativas	06 (11,8)
Doenças infecciosas e parasitárias	01 (2)
Outros	03 (5,9)
**CID da internação**	
J18 (Pneumonia)	11 (21,57)
K74 (Fibrose e cirrose hepáticas)	07 (13,73)
I50 (Insuficiência cardíaca)	06 (11,76)
I64 (Acidente vascular encefálico)	05 (9,8)
N17 (Insuficiência renal aguda)	05 (9,8)
**Tipo de internação**	
Clínica	50 (98)
Cirúrgica	01 (2)

A fraqueza muscular foi identificada como uma das principais circunstâncias, presente em 66,7% dos casos. A principal queixa entre os participantes internados foi a dispneia (21,6%), seguida por complicações de Acidente Vascular Encefálico (AVE), Insuficiência Cardíaca Congestiva (ICC) e Doença Renal Crônica (DRC), todos com taxa de 9,8%. Em relação ao motivo que levou à internação, categorizado por sistemas fisiológicos, as doenças do aparelho respiratório foram as mais prevalentes na amostra (33,3%), seguidas por doenças cardiovasculares (17,6%). O tipo de internação clínica foi predominante, abrangendo 98% dos casos. O diagnóstico principal da internação, de acordo com o CID-10, foi identificado nos prontuários dos pacientes, sendo mais comum a pneumonia de etiologia não especificada - J18 (21,6%) e a fibrose e cirrose hepáticas - K74 (13,73%).

### Escalas de avaliação

Ao avaliar a função cognitiva dos pacientes, observa-se uma média de 4,2 pontos (±2,7), predominando provável déficit cognitivo (62,7%). Para o grau de dependência em ABVD, a média foi de 3,6 pontos (±2,5), predominando independência (43,1%). Para o grau de dependência em AIVD, a média foi de 13,2 pontos (±5,2), predominando dependência parcial (74,5%) para este tipo de atividade. As informações detalhadas são apresentadas na Tabela 2.

### Associação entre faixa etária, declínio cognitivo e dependência funcional

Após a aplicação do teste exato de Fisher, foi possível identificar a existência ou não de associação entre a faixa etária e os desfechos, sendo considerados dois grupos: pessoas idosas com idade entre 60 e 79 anos (n=37) e idade igual ou superior a 80 anos (n=14). A análise demonstra associação estatística significativa, de moderada magnitude, entre a faixa etária e as variáveis função cognitiva (p<0,02), ABVD (p<0,01) e AIVD (p<0,03). Observa-se maior dependência para ABVD e pior desempenho cognitivo conforme o avançar da idade. Para as AIVD, foi identificada dependência parcial mais acentuada entre indivíduos com menos de 80 anos, além de um aumento gradual da dependência à medida que a idade avança. As informações detalhadas são apresentadas na Tabela 3.

**Tabela 3:** Associação entre a faixa etária e a função cognitiva e grau de dependência para realização de atividades de vida diária entre as pessoas idosas avaliadas, São Carlos, São Paulo, Brasil, 2025

Variáveis	Faixa etária		Valor de *p* ^*^
	60 a 79 anos (n=37)	≥80 anos (n=14)	
Desempenho cognitivo			<0,02
Função cognitiva normal	7 (18,9%)	0 (0%)
Possível déficit cognitivo	11 (29,7%)	01 (7,14%)
Provável déficit cognitivo	19 (51,3%)	13 (92,86%)
**Dependência em ABVD**			<0,01
Independente	21 (56,7%)	01 (7,1%)	
Parcialmente dependente	6 (16,2%)	03 (21,4%)	
Dependente	10 (27%)	10 (71,4%)	
**Dependência em AIVD**			
Independente	4 (10,8%)	05 (35,7%)	<0,03
Parcialmente dependente	31 (83,8%)	07 (50%)	
Dependente	2 (5,4%)	02 (14,3%)	

ABVD - Atividades Básicas de Vida Diária; AIVD - Atividades Instrumentas de Vida Diária;

* Teste exato de Fisher.

A estimação da razão de prevalência entre a variável faixa etária e os desfechos, realizada por meio do modelo de regressão de Poisson, é apresentada na Tabela 4. A associação entre faixa etária e ABVD revela que, entre os participantes com 80 anos ou mais, a prevalência de algum grau de dependência é aproximadamente duas vezes maior em comparação às pessoas idosas com menos de 80 anos. Em relação à avaliação cognitiva, estima-se que, em média, a prevalência de possível/provável déficit cognitivo seja 23% maior entre a faixa etária de 80 anos ou mais. Não foram observadas relações entre faixa etária e AIVD.

**Tabela 4:** Associações entre faixa etária e os desfechos recategorizados em modelo binário, São Carlos, São Paulo, Brasil, 2025

Variável	Faixa etária		Razão de prevalência^*^	Intervalo de confiança (95%)		Valor de *p*
	<80 anos (n=37)	≥80 anos (n=14)				
*Alguma dependência para ABVD*						
Não	21 (56,76%)	01 (7,14%)	2,15	1,44	3,19	<0,01
Sim	16 (43,24%)	13 (92,86%)				
*Alguma dependência para AIVD*						
Não	2 (5,41%)	02 (14,29%)	0,91	0,72	1,14	0,40
Sim	35 (94,59%)	12 (85,71%)				
*Provável ou possível déficit cognitivo*						
Não	7 (18,92%)	0 (0%)	1,23	1,06	1,44	0,01
Sim	30 (81,08%)	14 (100%)				

ABVD - Atividades Básicas de Vida Diária; AIVD - Atividades Instrumentas de Vida Diária;

* Estimado pelo modelo de regressão Poisson com variância robusta.

## DISCUSSÃO

Os achados deste estudo evidenciam uma predominância de pacientes do sexo feminino no setor de internação, corroborando os resultados de outros estudos realizados no âmbito da internação clínica^(15)^. Esse fenômeno pode ser atribuído à maior participação das mulheres em pesquisas relacionadas à saúde, decorrente de sua exposição mais frequente a tratamentos ao longo da vida e ao processo de feminização da velhice^(16)^. Em outros estudos conduzidos no contexto de internação hospitalar, observa-se predominância do sexo masculino. Essa tendência pode ser atribuída à propensão dos homens em buscar atendimento e serviços médicos de forma tardia, o que os torna mais vulneráveis à necessidade de internação devido à progressão e agravamento de suas condições de saúde^(17)^.

A idade média dos participantes foi de 73,6 anos, com 41% da amostra pertencendo ao contingente entre 60 e 69 anos. Estudos que investigam os comportamentos de saúde de pessoas idosas em ambiente de internação hospitalar relatam médias de idade variando entre 70 e 80 anos^(5,18)^. Já é estabelecido na literatura nacional que o maior número de internações, bem como a taxa de mortalidade, sofre aumento de forma concomitante com a faixa etária analisada^(19)^.

No presente estudo, a média de anos de escolaridade foi de 7,35, indicando um predomínio de baixa escolaridade entre os participantes, o que está em concordância com outros estudos que identificaram a baixa escolaridade entre pessoas idosas no contexto da internação hospitalar^(20,21)^. Baixos níveis de letramento em saúde estão associados a taxas mais elevadas de hospitalização e mortalidade, bem como a uma adesão deficiente aos regimes de tratamento e a uma redução na eficácia das medidas preventivas, impactando significativamente a qualidade de vida dessa população^(22)^. Esses fatores também estão relacionados à exclusão social, ao menor acesso a informações e a condições socioeconômicas desfavoráveis^(23)^. Essa situação pode ser atribuída à ausência de fatores protetivos, como a habilidade de ler e escrever, e à perda de informações importantes sobre saúde e autocuidado, o que favorece o acesso a informações falsas^(22)^.

No que diz respeito à ocupação atual e à fonte de renda, observa-se que a maioria dos participantes é aposentada (90,2%). Em revisão sistemática da literatura, Melo, Falsarella e Neri^(24)^ destacam a renda e a escolaridade como fatores primordiais associados à fragilidade em pessoas idosas. Dado o impacto desses fatores na qualidade de vida desses indivíduos, faz-se necessário o investimento público em estratégias eficazes, como a ampliação do acesso à educação formal, adaptada às necessidades da população idosa, visando à promoção de um envelhecimento saudável, à promoção da autonomia e à integração social, contribuindo para a saúde física e mental, reduzindo o isolamento social e aumentando o senso de propósito^(25)^.

Quanto às questões relacionadas à saúde, observa-se a presença de polipatologia em 60,8% da amostra. Um estudo conduzido por Tavares e Dias^(26)^, com uma amostra de 2.142 pessoas idosas, revelou que 98,3% dos participantes possuíam de quatro a sete condições de saúde distintas. A presença dessas condições pode dificultar o tratamento e aumentar significativamente o risco de complicações durante a internação, prolongando o período de hospitalização e elevando o risco de complicações pós-hospitalares, refletindo a necessidade de integrar diferentes níveis de atenção à saúde para garantir o acompanhamento desses indivíduos e a identificação precoce de agravos^(27)^. A literatura evidencia uma associação entre doenças crônicas, como câncer, diabetes mellitus, distúrbios cardiovasculares e doenças pulmonares, que, muitas vezes, levam ao uso excessivo de medicamentos e à necessidade de estratégias abrangentes para o manejo medicamentoso^(28,29)^.

A prevalência de polifarmácia foi observada em 62,7% da amostra, refletindo a tendência nacional de uso de cinco ou mais medicamentos diários^(30)^. Chang et al.^(31)^ conduziram um estudo abrangente com mais de 3 milhões de indivíduos acima dos 65 anos, no qual investigaram a correlação entre polifarmácia e os riscos de hospitalização e mortalidade. Os resultados demonstraram uma associação positiva crescente entre o número de medicamentos prescritos e a probabilidade de ocorrência de eventos adversos. Os efeitos negativos de regimes medicamentosos complexos sobre os desfechos clínicos foram evidenciados na investigação da relação entre polifarmácia, demência e mortalidade hospitalar em pacientes^(32)^. A polifarmácia, portanto, pode desencadear consequências severas, como quedas e deterioração cognitiva, frequentemente levando à hospitalização^(33)^.

A ocorrência de quedas nos últimos seis meses foi relatada por 58,8% da amostra, sendo a fraqueza muscular identificada como a principal causa (66,7%). A redução na capacidade funcional, combinada com alterações fisiológicas associadas ao envelhecimento, aumenta a prevalência de quedas nessa população^(34)^. Além disso, o uso de medicamentos é considerado um fator de risco para quedas, especialmente devido à polifarmácia, em consequência das interações medicamentosas^(35)^. De acordo com Sugitani e Ito^(36)^, medicamentos como diuréticos e anti-hipertensivos, frequentemente prescritos para o tratamento da hipertensão arterial sistêmica, estão associados ao aumento do risco de quedas devido à hipotensão postural e vertigem. Além disso, psicotrópicos, incluindo benzodiazepínicos, antidepressivos e antipsicóticos, contribuem para o risco de quedas devido aos seus efeitos sedativos e comprometimento do equilíbrio^(37)^. Por isso, é fundamental promover a educação em saúde para pacientes e seus cuidadores, visando garantir o uso seguro dos medicamentos.

No que diz respeito à avaliação cognitiva, constatou-se um predomínio de pacientes com provável déficit cognitivo (62,7%). O declínio cognitivo é um processo que envolve alterações nas capacidades intelectuais e, com sua progressão, pessoas idosas enfrentam dificuldades na execução de ABVD e AIVD, o que afeta negativamente sua autonomia, contribui para o aumento dos índices de institucionalização e eleva o risco de fragilidade^(38)^. De acordo com Cunha et al.^(39)^, 35% dos idosos sofrem um declínio na capacidade cognitiva quando hospitalizados. O ambiente hospitalar pode desencadear ou intensificar o declínio cognitivo nessa população devido a fatores como polifarmácia, infecções, multimorbidades, privação de estímulos sensoriais e mudanças nos hábitos cotidianos^(7)^.

Pessoas idosas com baixo nível de escolaridade apresentam uma probabilidade de 4,5 a 5 vezes maior de desenvolver algum tipo de comprometimento cognitivo em comparação àquelas com níveis educacionais mais elevados^(7).^ Além disso, idosos analfabetos têm uma suscetibilidade cinco vezes maior à dependência funcional. Esse elevado número reflete a falta de acesso ao sistema educacional no passado, o que impacta significativamente o perfil cognitivo dessa população atualmente^(40)^.

No presente estudo, observou-se uma correlação significativa entre a função cognitiva e a idade. Estima-se que, em média, entre pessoas idosas com 80 anos ou mais, a prevalência de provável ou possível déficit cognitivo seja 23% maior em comparação àquelas com menos de 80 anos^(41)^. Quando associado à idade avançada e ao período de hospitalização, Covinsky et al.^(42)^ demonstraram que mais de 35% dos participantes (2.761 com idade igual ou superior a 70 anos) tiveram piora em sua capacidade funcional. Cunha et al.^(39)^ argumentam que a idade não é tão relevante quanto a piora clínica, que é o principal fator de interesse, pois contribui para a progressão do declínio cognitivo e impacta na autonomia dessa população.

É crucial que a equipe multiprofissional nos serviços de saúde, especialmente nas áreas de internação, implemente estratégias para a avaliação cognitiva dos pacientes, empregando instrumentos específicos, buscando identificar precocemente sinais de declínio cognitivo e prevenir a perda de autonomia, uma vez que a qualidade de vida desses indivíduos está intimamente ligada ao nível de independência na realização de atividades diárias^(43)^. Em relação à autonomia para a realização das ABVD, 39,22% da amostra apresentou dependência, corroborando os achados de outras pesquisas^(44)^.

A idade dos participantes e o grau de dependência para ABVD mostraram-se estatisticamente relacionados. Diversos fatores podem contribuir para a perda de capacidade em pessoas idosas hospitalizadas, como o desenvolvimento de infecções, o tempo prolongado de internação e o declínio cognitivo. Além disso, a restrição ao leito provoca mudanças na musculatura e na massa óssea, resultando em limitações na execução das atividades diárias^(45)^. Um estudo realizado com pessoas idosas internadas no Hospital das Clínicas da Faculdade de Medicina de Botucatu identificou que a funcionalidade da amostra na alta hospitalar era inferior à observada antes da internação^(23)^. As perdas funcionais em ABVD são quase o dobro em relação às AIVD, sugerindo que as primeiras funcionam como marcadores sensíveis para avaliar a socialização dessa população. Dessa forma, se o indivíduo enfrenta dificuldades em alguma atividade de vida diária, é provável que também apresente dificuldades em duas ou mais AIVD^(46)^.

Para as AIVD, 74,5% da amostra apresentou dependência parcial em relação a esse tipo de atividade. Em um estudo com 94 idosos internados em uma clínica médica, constatou-se que, apesar da autonomia preservada, os participantes apresentaram certo grau de dependência tanto nas ABVD quanto nas AIVD, indicando algum comprometimento da independência. A maioria da amostra foi avaliada ainda na primeira semana de internação, sugerindo que o estado funcional dos pacientes já estava comprometido antes da hospitalização, possivelmente devido à exposição a fatores de risco, como a idade avançada^(44)^.

Para que o envelhecimento seja bem-sucedido, é crucial que as pessoas idosas mantenham, além de uma interação multidimensional adequada entre a saúde física, mental e a integração social, a independência nas atividades diárias. A equipe de saúde desempenha um papel vital na manutenção da funcionalidade global dessa população, tanto na identificação precoce do declínio funcional quanto na implementação de ações para preservar a autonomia no período pós-hospitalização^(47)^. Isso inclui, por exemplo, um plano de alta hospitalar responsável e o acompanhamento contínuo em outros níveis de atenção à saúde.

A implementação de ações direcionadas à preservação da funcionalidade e cognição da população idosa é fundamental no contexto de internação hospitalar. Para tanto, é essencial que os profissionais de enfermagem detenham domínio teórico-prático sobre as alterações fisiológicas do envelhecimento, permitindo não apenas a identificação precoce de desvios, mas também a execução de intervenções assertivas^(48)^. Destaca-se a necessidade de os enfermeiros promoverem a capacitação contínua da equipe, assegurando assistência pautada em sensibilidade, segurança e competência técnica. Isso inclui o desenvolvimento de protocolos padronizados para avaliação do declínio funcional, aliados a ações direcionadas que englobem tanto a detecção precoce quanto o manejo especializado dessa população.

Entre as estratégias mais eficazes, destaca-se a avaliação contínua do estado funcional dos pacientes, uma vez que essa preservação está intimamente ligada à redução do risco de mortalidade em pessoas idosas hospitalizadas^(48)^. As intervenções, como a promoção de exercícios físicos adaptados, o estímulo à mobilidade, a estimulação cognitiva, o manejo adequado de medicamentos e o fornecimento de cuidados individualizados, são exemplos de abordagens conduzidas por equipes multidisciplinares no ambiente hospitalar, que demonstraram resultados positivos e impacto significativo na manutenção desses fatores^(49)^.

### Limitações do estudo

Os resultados deste estudo devem ser analisados à luz de algumas limitações. A coleta de dados realizada à beira do leito apresentou desafios importantes. Embora essa metodologia ofereça a vantagem de um contato direto e imediato com o paciente e sua realidade clínica, surgiram dificuldades associadas à baixa escolaridade, déficits cognitivos graves e condições clínicas debilitantes. Além disso, a impossibilidade de alguns participantes de se comunicarem, somada à falta de um cuidador ou familiar para auxiliá-los, também impossibilitou a continuidade da coleta de dados em alguns momentos. Ademais, o ambiente hospitalar, por sua natureza dinâmica, está sujeito a emergências, interrupções e variações no estado de saúde dos pacientes.

### Contribuições para a área da Enfermagem, Saúde ou Política Pública

Diante das mudanças demográficas e epidemiológicas, caracterizadas pelo aumento de DCNTs e multimorbidades, os profissionais de enfermagem assumem papel central não apenas na assistência direta, mas também na identificação de padrões de internação e na articulação de estratégias que respondam às complexidades desse cenário. A alta prevalência de polipatologia e polifarmácia observada demonstra a necessidade de estratégias de manejo clínico mais complexas, nas quais a enfermagem atua como mediadora entre as demandas biológicas, sociais e educacionais. A gestão segura de múltiplos medicamentos, a prevenção de interações adversas e a educação de pacientes e familiares sobre adesão terapêutica são competências que demandam protocolos clínicos especializados e uma abordagem interdisciplinar. Além disso, fatores socioeconômicos, como acesso limitado a serviços de saúde e baixa escolaridade, exigem que a enfermagem incorpore práticas culturalmente sensíveis, garantindo que as intervenções transcendam o ambiente hospitalar e se alinhem às realidades vividas pelos pacientes.

O declínio cognitivo e a dependência funcional observados sublinham a importância de estratégias de reabilitação para manter a autonomia e a qualidade de vida da população idosa no momento de internação, reforçando a urgência de estratégias de reabilitação precoces durante esse período. A promoção de mobilização precoce, o estímulo à participação nas atividades de vida diária e a adaptação do ambiente hospitalar às necessidades funcionais das pessoas são medidas essenciais para preservar sua qualidade de vida e reduzir complicações associadas à imobilização prolongada e devem ser implementadas diariamente pela equipe de enfermagem.

A população idosa representa um importante contingente do total de internações hospitalares, por isso, identificar o perfil de internação e os tipos de relação aqui evidenciados possibilita o desenvolvimento de novas estratégias e perspectivas de cuidado, contribuindo para a formulação de novas políticas de saúde e para o desenvolvimento de protocolos de saúde baseados em evidências, considerando as demandas impostas pela mudança no cenário demográfico e epidemiológico brasileiro.

## CONCLUSÕES

Os achados revelam predominância de participantes com provável déficit cognitivo, independência para ABVD e dependência parcial para AIVD. A relação entre declínio cognitivo e dependência funcional para atividades de vida diária com o avançar da idade evidencia a importância de intervenções e avaliações precoces e recorrentes. É fundamental a implementação de estratégias que visem à manutenção da funcionalidade, à prevenção de declínio cognitivo e ao suporte psicossocial, tanto durante a internação quanto no planejamento da alta hospitalar, por meio de uma rede estruturada que possibilite o acompanhamento dessa população em diferentes níveis de atenção à saúde, além da capacitação constante da equipe multiprofissional e de cuidadores, contribuindo para a diminuição das taxas de reinternações.

## Data Availability

Os dados da pesquisa estão disponíveis no corpo do artigo.
